# Transfemoral Valve-in-Valve Transcatheter Aortic Valve Replacement in a Patient With Chronic Dissection Flap

**DOI:** 10.1016/j.jscai.2026.105443

**Published:** 2026-06-02

**Authors:** Stevan S. Pupovac, Caleb A. Sokolowski, James R. Taylor, Alan R. Hartman, Elana Koss, Robert S. Palazzo, Bruce Rutkin

**Affiliations:** Department of Cardiothoracic Surgery, Northwell Health, Manhasset, New York

**Keywords:** aortic dissection, transcatheter aortic valve replacement, valve-in-valve

## Introduction

Transcatheter aortic valve-in-valve implantation is an established therapy for failed surgical bioprosthetic valves and may obviate the need for reoperative surgery. Despite the routine use of large-bore transfemoral access in endovascular aortic repair, its application in structural heart interventions involving chronic thoracic aortic dissection remains uncommon.^1^ To date, only 2 cases of transcatheter aortic valve replacement in patients with a chronic descending thoracic aortic dissection have been described.^2^^,^^3^ We report a case of transfemoral valve-in-valve transcatheter aortic valve replacement in this setting.

## Case presentation

**Figure 1:**
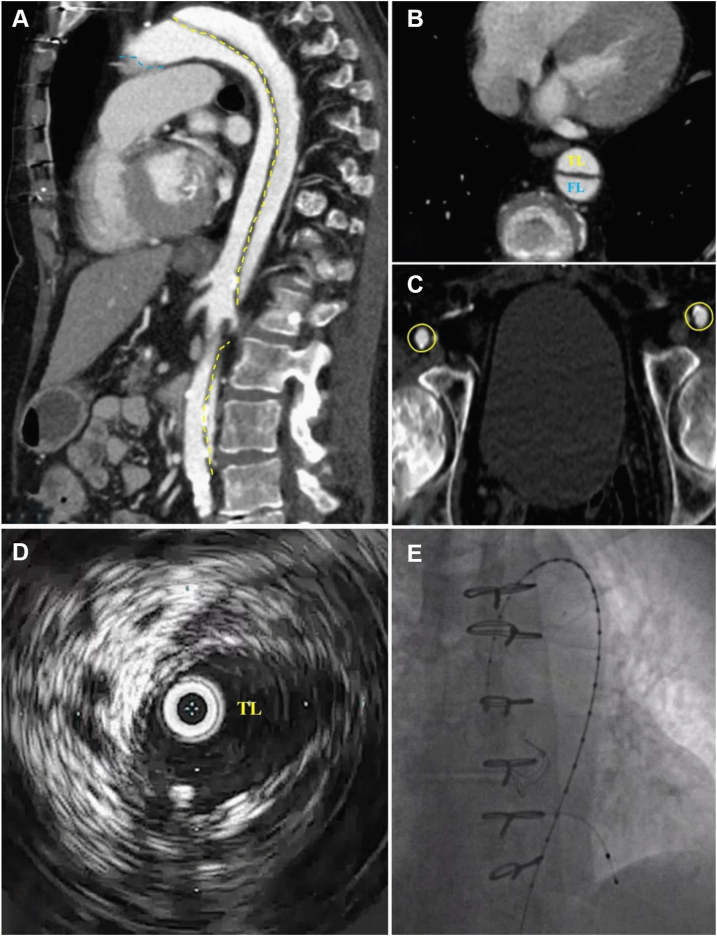
**Preprocedural computed tomography assessment of chronic descending thoracic aortic dissection and intraoperative intravascular ultrasound–guided confirmation of true lumen access.** (A) Curved planar reconstruction demonstrating the distal surgical anastomosis (blue dotted line) and a chronic descending thoracic aortic dissection with a noncircumferential intimal flap extending distally (yellow dotted line). (B) Axial computed tomography image of the descending thoracic aorta demonstrating preserved true lumen (TL) adjacent to the false lumen (FL). (C) Axial image at the level of the common femoral arteries demonstrating preserved, nondissected bilateral common femoral artery anatomy suitable for large-bore transfemoral access. (D) Intravascular ultrasound image demonstrating the catheter positioned within the TL during transfemoral access across a chronic descending thoracic aortic dissection. (E) Fluoroscopic image demonstrating intravascular ultrasound catheter advancement through the descending thoracic aorta. FL, false lumen; TL, true lumen.

An 84-year-old woman with severe obstructive pulmonary disease requiring chronic steroid therapy and tracheobronchomalacia presented with symptomatic severe bioprosthetic aortic stenosis 12 months after aortic root and hemiarch replacement for acute DeBakey type I aortic dissection. A residual chronic dissection flap extended from the distal anastomosis through the descending thoracic aorta to the aortoiliac bifurcation (Figure 1A-C).

Given prohibitive surgical risk, a transcatheter approach was pursued. Alternative access strategies were considered; however, given the patient’s significant pulmonary comorbidities and the presence of a chronic descending thoracic aortic dissection, transfemoral access was favored following multidisciplinary heart team discussion.

Cardiac-gated computed tomography demonstrated a chronic dissection originating at the distal anastomotic site with preservation of bilateral common femoral artery integrity. Valve selection was guided by early degeneration of the prior bioprosthesis and the need to optimize effective orifice area within a small (21 mm) surgical valve, favoring a supra-annular transcatheter platform.

The procedure was performed under monitored anesthesia care. Percutaneous bilateral common femoral artery access was obtained, and intravascular ultrasound was used to confirm true lumen cannulation and stable wire passage through the descending thoracic aorta into the ascending aortic graft (Figure 1D-E). A valve-in-valve transcatheter aortic valve was advanced and deployed without complication.

Postdilation was performed to optimize valve expansion. Completion echocardiography demonstrated no paravalvular regurgitation and a mean transvalvular gradient of 6 mm Hg. The patient was discharged home and had improvement of her symptoms at outpatient follow-up.

### Comment

To our knowledge, this is the first reported case of valve-in-valve transcatheter aortic valve replacement performed via percutaneous transfemoral access in a patient with a chronic descending thoracic aortic dissection. Although prior reports have described transcatheter aortic valve replacement in the setting of chronic aortic dissection using transfemoral or alternative access strategies, experience remains limited.

In the present case, transfemoral access was selected after multidisciplinary heart team evaluation, as alternative access strategies were felt to confer greater risk given the patient’s comorbid conditions. As the population of patients with prior complex aortic surgery expands, scenarios requiring transcatheter valve intervention in the setting of chronic aortic dissection may be encountered more frequently.^4^ This case demonstrates the technical feasibility of a transfemoral valve-in-valve transcatheter aortic valve replacement strategy in a carefully selected patient guided by detailed imaging and procedural planning.

## Conclusion

Transfemoral valve-in-valve transcatheter aortic valve replacement is technically feasible in selected patients with a chronic descending thoracic aortic dissection when guided by careful imaging assessment, including intravascular ultrasound.

## CRediT authorship contribution statement

**Stevan S. Pupovac:** Writing – original draft, Investigation, Conceptualization. **Caleb A. Sokolowski:** Writing – review & editing, Writing – original draft, Conceptualization. **James R. Taylor:** Visualization, Supervision, Project administration. **Alan R. Hartman:** Visualization, Software, Resources, Investigation. **Elana Koss:** Visualization, Investigation, Data curation. **Robert S. Palazzo:** Visualization, Validation, Supervision, Data curation. **Bruce Rutkin:** Writing – review & editing, Visualization, Supervision, Resources, Formal analysis, Conceptualization.
